# Viburnum Prunifolium in Threatened Abortion and in Menorrhagia

**Published:** 1879-02-20

**Authors:** D. B. Nisbet

**Affiliations:** Georgia


### *VIBURNUM PRUNIFOLIUM IN THREATENED ABORTION AND IN MENORRHAGIA.*

BY D. B. NISBET, M. D., OF GEORGIA.

I send you report of two cases in which tincture viburnum was used with success; you can publish same if you deem it necessary.

*Case 1st.* On September 23d, 1878, I was summoned in haste to see Morilla ——, a colored woman about 32 years old, who had received a wound from a fall. On reaching her home I learned that patient, while attempting to replace a rope in a well whirl, had fallen from the well upon the sharp-ragged edge of a rail which was driven in the ground near by, with one end protruding, making a wound about five inches long, just above pubes, penetrating as far as the muscles of the abdomen. The woman being somewhat advanced in pregnancy, the fall brought on strong labor pains.

After dressing the wound with ligature and adhesive strips, I made examination per vaginam, found the os considerably dilated, pains continuing regularly. I gave her:

Tinct. viburnum .............. ʒj

Water.......................... ozj with direction to take the same quantity if the pains did no cease in one hour.

Calling two or three hours after, I found the patient resting very quietly, with pains relieved. Woman recoverd, and will soon be confined.

*Case 2d.* The next case was a case of menorrhagia (excessive menstruation), in which the various preparations of iron, ergot, etc., were used without relief. I finally put patient on a teaspoonful tinct. viburnum, beginning with the medicine two days before her period. Flow was considerably diminished. Ordered her to repeat same at her next period, which she did with marked relief. Patient was a married woman; had been married two years; no children. After taking the viburnum a second time she became pregnant and will soon have reached full term.

The medicine acts, no doubt, as a uterine sedative, but I cannot explain the action. I would be very glad if I could see something in reference to it in your valuable journal.

